# Mitigating disrespect and abuse during childbirth in Tanzania: an exploratory study of the effects of two facility-based interventions in a large public hospital

**DOI:** 10.1186/s12978-016-0187-z

**Published:** 2016-07-18

**Authors:** Hannah L. Ratcliffe, David Sando, Goodluck Willey Lyatuu, Faida Emil, Mary Mwanyika-Sando, Guerino Chalamilla, Ana Langer, Kathleen P. McDonald

**Affiliations:** Women and Health Initiative, Department of Global Health and Population, Harvard T.H Chan School of Public Health, Boston, MA USA; Ariadne Labs at Brigham and Women’s Hospital and the Harvard T.H. Chan School of Public Health, Boston, MA USA; Management and Development for Health, Dar es Salaam, Tanzania; Africa Academy for Public Health, Dar es Salaam, Tanzania; Boston University School of Public Health, Boston, MA USA

**Keywords:** Maternal health, Disrespect and abuse, Respectful maternity care, Tanzania, Health workers for change, Quality of care, Quality improvement

## Abstract

**Background:**

There is emerging evidence that disrespect and abuse (D&A) during facility-based childbirth is prevalent in countries throughout the world and a barrier to achieving good maternal health outcomes. However, much work remains in the identification of effective interventions to prevent and eliminate D&A during facility-based childbirth. This paper describes an exploratory study conducted in a large referral hospital in Dar es Salaam, Tanzania that sought to measure D&A, introduce a package of interventions to reduce its incidence, and evaluate their effectiveness.

**Methods:**

After extensive consultation with critical constituencies, two discrete interventions were implemented: (1) Open Birth Days (OBD), a birth preparedness and antenatal care education program, and (2) a workshop for healthcare providers based on the Health Workers for Change curriculum. Each intervention was designed to increase knowledge of patient rights and birth preparedness; increase and improve patient-provider and provider-administrator communication; and improve women’s experience and provider attitudes. The effects of the interventions were assessed using a pre-post design and a range of tools: pre-post questionnaires for OBD participants and pre-post questionnaires for workshop participants; structured interviews with healthcare providers and administrators; structured interviews with women who gave birth at the study facility; and direct observations of patient-provider interactions during labor and delivery.

**Results:**

Comparisons before and after the interventions showed an increase in patient and provider knowledge of user rights across multiple dimensions, as well as women’s knowledge of the labor and delivery process. Women reported feeling better prepared for delivery and provider attitudes towards them improved, with providers reporting higher levels of empathy for the women they serve and better interpersonal relationships. Patients and providers reported improved communication, which direct observations confirmed. Additionally, women reported feeling more empowered and confident during delivery. Provider job satisfaction increased substantially from baseline levels, as did user reports of satisfaction and perceptions of care quality.

**Conclusions:**

Collectively, the outcomes of this study indicate that the tested interventions have the potential to be successful in promoting outcomes that are prerequisite to reducing disrespect and abuse. However, a more rigorous evaluation is needed to determine the full impact of these interventions.

## Background

In recent years, the maternal health community has increasingly focused on quality of care as a critical component of efforts to improve maternal health outcomes and reduce maternal mortality. In this context, the importance of eliminating disrespect and abuse (D&A) during facility-based childbirth has received growing attention. Disrespect and abuse is a fundamental violation of women’s human rights [1], and evidence is mounting that D&A may undermine women’s trust in the health system and deter them from seeking facility-based care for delivery [2–4].

Bowser and Hill [5] categorized disrespect and abuse into seven dimensions: physical abuse, non-consented care, non-confidential care, non-dignified care, discrimination, abandonment of care, and detention in healthcare facilities. Since then, much work has been done to refine these definitions [6–10]. D&A is a complex issue that can be interpreted differently by women, their families, healthcare providers, and administrators [7]. Importantly, D&A can be the result of both structural and interpersonal factors [8]. At the structural level, inadequate facility infrastructure may interfere with privacy during labor and delivery, and poor supervision and management may foster unaccountable work environments [5, 11]. At the same time, individual provider biases and discrimination may result in disrespectful care [5]. Health providers themselves may also be subject to disrespectful treatment on the part of the health system, which can lead to burnout and further disrespect and abuse of patients [12–14]. These factors reinforce a vicious cycle that perpetuates the pattern of abuse. Although work remains to further refine and operationalize the definition of D&A, it is clear that D&A indicates health systems in crisis [15].

Significant work has been conducted in recent years to quantify the prevalence of disrespect and abuse in a variety of healthcare settings, and emerging evidence from many countries and contexts has demonstrated that D&A is a widespread problem [15–18]. A recent review found three studies which sought to estimate the prevalence of D&A, with results ranging from 15 to 98 % [8]. However, despite the evidence that demonstrates that D&A is common, few studies have been conducted to identify and evaluate interventions with the potential to ameliorate D&A and promote respectful maternity care (RMC) [19]. More evidence about interventions with the potential to mitigate the drivers and manifestations of D&A is critically needed [20], along with a better understanding of the processes that lead to these changes [10].

This paper describes the implementation process and outcomes of two interventions to reduce disrespect and abuse in a large referral hospital in Dar es Salaam, Tanzania. By making explicit our theory of change and highlighting specific changes over time, the implementation strategy described here provides valuable process information that may inform the design of future intervention and evaluation efforts. The work described is part of a larger implementation research project focused on measuring the prevalence of D&A, describing its contributory factors, and testing methods of mitigating its occurrence. Based on the findings of a baseline assessment, a package of interventions was designed and implemented to address the key factors identified as driving disrespect and abuse at the study facility. This paper describes in detail the process undertaken to select and implement a package of interventions to address the concerns identified, as well as preliminary outcomes of the interventions on patients, providers, and the health facility.

## Methods

### Design

This project was conducted between January 2013 and December 2014. We organized the activities in three phases, preceded by several sensitization meetings with key stakeholders at the national, district, and facility levels. Phase one of the project was the baseline study (Sando D, Ratcliffe H, McDonald K, Spiegelman D, Lyatuu G, Mwanyika-Sando M. The prevalence of disrespect and abuse during facilitybased childbirth in urban Tanzania, Forthcoming). Phase two focused on intervention selection and implementation. The two interventions chosen—Respectful Maternity Care Workshops and Open Birth Days—were implemented over a seven-month period. The evaluation—phase three of the project—was conducted concurrently with the intervention phase and during the last six months of the project and used a before and after design.

### Setting

The study was conducted at a large, urban regional referral hospital in Dar es Salaam, Tanzania. The hospital has a catchment area of 1.4 million people and the maternity section of the facility serves as a referral site to over 40 lower level health facilities [21]. During the implementation and evaluation phases, the study facility averaged 2060 deliveries, 311 maternal complications, 3 maternal deaths, and 68 neonatal deaths per month (Facility data). Due to crowded conditions, women are not allowed a birth companion during labor and delivery at this facility.

### Implementation

#### Baseline

A baseline assessment was conducted from April and October 2013 and the results are published elsewhere (Sando, et al., The prevalence of disrespect and abuse during facility-based childbirth in urban Tanzania, Forthcoming). The methods employed in the assessment are detailed in Table 1. 2000 women were interviewed at the hospital three to six hours postpartum. Of those, 15 % reported experiencing any category of disrespect and abuse. A sub-sample of 77 women were re-interviewed in their homes four to six weeks after birth. Among them, the reported prevalence of experienced D&A increased to 77 %. In addition to the categories of D&A defined by Bowser and Hill, respondents also reported high levels of “lack of information.” Additionally, 200 direct observations of interactions between women and providers during labor and delivery were conducted. These recorded very high prevalence of some instances of D&A, including over 80 % of women not being asked for consent during examinations, 60 % of women not being covered during delivery, and over 90 % of women having to share a bed with other women in the postnatal ward. Finally, structured interviews with 50 providers found that providers had little knowledge of patients’ rights while reporting low levels of job satisfaction.

**Table 1:** Baseline assessment methods and sample size

Methods	Description	Number
Postpartum interviews	Women who gave birth at the facility were interviewed about their care experience at their time of discharge, approximately three to six hours post-delivery. Every second woman entering the postnatal ward was systematically sampled for inclusion.	2000
Direct observation	Trained nurse-midwives conducted observations of patient-provider interactions throughout a woman’s stay at the facility, from admission to 2 h postpartum. Observation was stopped if the woman was transferred to the operating theatre for a Caesarean section. Women presenting at the registration desk for delivery were systematically sampled for inclusion. 100 women were both directly observed and interviewed in the postpartum interview.	208
Community follow-up interviews	Attempts were made to follow-up with all 100 women who were both observed during labor and delivery and interviewed postpartum. 70 were contacted and agreed to participate. Interviews were conducted in women’s homes four to six weeks after delivery.	70
Structured provider interviews	All providers and administrators working in the maternity block at the study facility were interviewed about their job satisfaction and perceptions of quality of care.	50
In-depth provider interviews	Providers working in the maternity block and hospital and municipal administrators completed semi-structured in-depth interviews.	18

#### Selection of interventions

Following the completion of the baseline study, a three-part, stepwise dissemination and participatory intervention selection process was undertaken with providers and administrators at the study facility, district officials, and national representatives. Through this iterative dissemination process, two discrete interventions—Open Birth Days and a Respectful Maternity Care Workshop—were selected by a multi-stakeholder working group based on the hypothesis that the interventions would have a positive cumulative impact on the knowledge and attitudes of and communication between patients and providers. Stakeholders deemed the interventions to be feasible, acceptable and sustainable in the existing resource-constrained health system.

#### Implementation of interventions

Open Birth Days (OBD) were designed to address two salient needs identified from the baseline data: (1) provider complaints that women were inadequately prepared for delivery, including not knowing where to go, what to bring, what to expect, or what their rights and responsibilities as patients were; and (2) patients’ reports that they lacked basic information about the labor and delivery process. OBD complemented the antenatal care offered at the hospital (i.e., Focused Antenatal Care (FANC)). All women attending FANC at the study hospital who were in their third trimester of pregnancy were eligible to participate. Nurses providing antenatal care at the facility identified all eligible women at the beginning of every clinic session, explained to them about OBD, and invited them to stay after their clinic session to participate.

OBD sessions were implemented from May to October 2014. OBD were designed to facilitate communication between women and providers, offer women a step-by-step list of what to expect when arriving at the hospital for delivery, provide guidance to women on which commodities they should bring with them, empower women to advocate for quality health care by orienting them on their rights during childbirth, and generate trust and mutual accountability between providers and patients by openly discussing expectations of and for both groups. To facilitate this discussion on patient rights, the *Universal Rights of Childbearing Women* (“the Charter”) was translated into Kiswahili in collaboration with the White Ribbon Alliance [1]. Poster-size copies of the Charter were created and hung in all wards on the maternity block, notebook-size copies were distributed to all staff to keep at their workspace, and postcard-size copies were distributed to all women attending OBD.

OBD consisted of a participatory health education session and a tour of the hospital that included all the wards that women might encounter during childbirth: registration, antenatal ward, labor and delivery ward, postnatal ward, pharmacy, operating theater, and the complaints department. Between May and October 2014, all 362 eligible women attending FANC at the study facility participated in an OBD Session (100 % acceptance rate).

The Respectful Maternity Care Workshop responded to provider and administrator requests for training in the area of RMC. The World Health Organization’s Health Workers for Change curriculum [22], which consists of six modules, was adapted as the basis for the RMC Workshop. Health Workers for Change has been evaluated in many different country and health system contexts and found to consistently promote open dialogue, improve patient-provider relationships, improve internal communication at facilities, and encourage self-reflection and increased empathy amongst providers [23–25]. The goal of the workshop was for providers to revisit their professional codes of conduct, assess their current practice in relation to these ethical principles, reflect on the personal situations of patients at the facility and what they may desire when they come to seek care, and openly and honestly reflect on interpersonal and structural barriers that prevent the provision of RMC at the study facility.

The RMC Workshop was facilitated by respected medical school professors and experienced quality improvement facilitators who completed a two-day orientation on the curriculum. Eighty-eight staff from the maternity ward—including all staff who provided care during labor, delivery, and the postpartum period—were approached to take part in the RMC Workshop, and 76 were willing and able to participate (86 % acceptance rate). Participants came from all units within the hospital maternity block, the Hospital Management Team, and the Council Health Management Team (district-level administration). Workshops were held throughout April and May 2014. Providers and administrators from the study facility were divided into five groups of 15–20 and all of them completed the six sessions over a period of two days per group. Senior facility and municipal administrators participated in the program, but were contained to a separate group so that frontline health workers in the other groups could engage in free and open discussion. Participants were asked to reflect on the barriers to providing respectful care—both interpersonal and structural—that they face in their daily work. At the conclusion of the workshop, participants were tasked with developing an action plan to address these issues.

### Evaluation

**Fig. 1:**
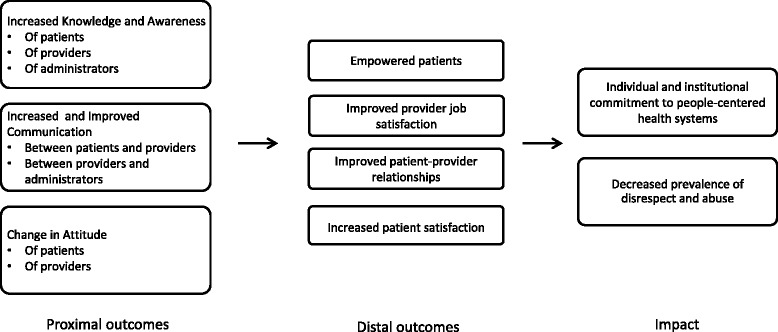
Theory of Change

A theory of change, specifying concrete outcomes, was used to guide intervention activities and evaluation efforts (Fig. 1). Multiple methods were applied to assess post intervention changes (Table 2). Pre- and post-tests with participants in OBD and pre- and post-tests with participants in the RMC Workshop were administered. Additionally, study staff conducted periodic observations of OBD sessions and short open-ended interviews with participants, as well as regular monitoring of progress towards achievement of the RMC Workshop action plan. During the evaluation, 459 direct observations of patient-provider interactions during labor and delivery were conducted by trained nurse-midwives, who were unaffiliated with the study facility. Every second woman registering at the facility for labor and delivery was selected for observation and provided informed consent. Additionally, women who had attended an Open Birth Day session (as designated by a sticker on their antenatal care card) were purposefully selected for observation. Structured community follow-up interviews were conducted four to six weeks postpartum with 149 women who gave birth at the study facility. Participants were selected systematically from both the Open Birth Days register and the list of women who were directly observed during labor and delivery. Women were contacted by phone to set up an interview time and provided informed consent at the time of interview. Finally, all providers and administrators working in the maternity block were interviewed using a structured interview tool (55 total, 72 % response rate).

**Table 2:** Monitoring and evaluation methods

	Methods	Description	Number
Monitoring Methods	Open Birth Days observations and brief interviews	To determine the acceptability of the Open Birth Days intervention, study staff conducted periodic observations of OBD sessions and conducted brief interviews with participants.	22 interviews
	Pre and Post-Tests for Open Birth Days	Women who attended OBD completed a short survey immediately before and after their OBD session to measure changes in knowledge about the labor and delivery process and their rights as patients. In addition, women were asked about their level of comfort with their future birth at the facility.	362
	Pre and Post-Tests for RMC Workshop	Participants in the RMC Workshop completed short surveys immediately before and after the workshop to assess changes in their knowledge of patients’ rights, views towards patients, and attitudes towards their jobs.	76
	RMC Workshop Action Plan	Progress towards implementation of the action plan developed at the end of the RMC Workshop was regularly monitored by study staff through discussions with key facility staff, including the head of the obstetrics and gynecology department and the Nurse Matron of the maternity block.	Monthly monitoring, 8 times from April-October
Evaluation Methods	Direct observation	A team of trained nurse-midwife observers rotated for twenty-four hour coverage at the registration desk of the study facility. Every second woman registering for labor and delivery was selected for observation. The observer followed the woman from the time of registration to two hours post-delivery or until transfer to the postnatal ward if this occurred after two hours. Women who presented at the study facility who had attended OBD were purposefully selected for observation and 57 women who attended OBD were observed. All observers were unaffiliated with the study facility.	459, including 57 women who attended OBD
	Community follow-up interviews	Interviews with women who gave birth at the facility were conducted four to six weeks post-delivery in the woman’s home. Women were selected for interview systematically from both the Open Birth Days register and the list of women who were directly observed during labor and delivery. Interviews were conducted by trained social scientists. The tool used from the baseline assessment was used and additional questions specific to OBD participation were included.	149, including 28 women who attended OBD
	Structured provider interviews	Providers and administrators working in the maternity block at the study facility were interviewed about their job satisfaction and perceptions of quality of care. Interviews were conducted in a private room at the facility by trained social scientists. Questions from the baseline assessment were replicated, and modules on the two discrete interventions (RMC Workshop and OBD) were added.	55, including 25 who participated in the RMC Workshop

### Analysis

Data analysis was performed using STATA Version 13. Categorical variables were summarized by proportion and continuous variables were summarized by mean and standard deviation. Outcomes of interest included: providers’ and women’s knowledge of patient rights, women’s knowledge of the labor and delivery process, provider and patient attitudes towards each other, provider-patient communication, women’s experiences of labor and delivery, patient satisfaction with care received, provider job satisfaction, and the quality of patient-provider relationships.

## Results

### Demographics

The mean age of women who were interviewed during community follow-up interviews was 29.7 years. Approximately 10 % were HIV positive and 17.5 % were nulliparous. The majority were married (82.6 %), had at least a primary education (82.6 %), and had completed at least four antenatal care visits for their most recent delivery (69.1 %). Overall, these demographic characteristics were similar to those of women interviewed during the baseline assessment, except for age (women interviewed for the evaluation tended to be slightly older than women at baseline: 29.7 vs. 25.2 years old). Also, some characteristics of women’s reproductive history in the baseline and evaluation groups differed, such as parity (women at baseline were more frequently nulliparous :36 % compared to 17.5 %), and coverage of antenatal care (57 % attended at least four compared to 69 % at evaluation) (Sando, et al. The prevalence of disrespect and abuse during facility-based childbirth in urban Tanzania, Forthcoming). Amongst women who were observed during labor and delivery, approximately half had one or two previous deliveries and 7 % were HIV positive.

The mean age of providers interviewed was 35.2 years, and 89 % were female. Approximately 80 % of respondents were nurses/nurse-midwives and 14.5 % included other clinical staff, such as medical officers and assistant medical officers. All wards of the maternity block, including antenatal (18 %), labor (36 %), and postnatal (33 %) were represented in our population and the median time respondents had spent working in their respective ward was one year.

### Intervention outputs

#### Open Birth Days

This intervention showed high acceptability among both women and providers. All women who were invited to participate in OBD consented. During the interviews conducted by study staff immediately after OBD sessions, all respondents expressed satisfaction with the intervention, with several commenting that it should be expanded to all other delivery facilities in Dar es Salaam. Among community follow-up interview respondents who had participated in OBD, 92.6 % said that they found the session “very helpful” and 96.3 % said they would be “very likely” to recommend to other women in their community to attend an OBD session before they deliver.

We collected feedback from health care providers midway through the implementation period, including the nurse in-charge of the reproductive and child health clinic, the matron of the maternity block, and the head of obstetrics and gynecology. All informants indicated that they were satisfied with OBD and noted that the intervention was manageable with their other duties and that they liked the opportunity to interact with their patients. Importantly, hospital staff said that the information provided during OBD was useful to both women and providers, particularly in reinforcing ethical codes and principles and facilitating more informal interactions between women and providers.

#### Respectful maternity care workshop

Staff perceptions of the workshop were positive. Observations by study team members and feedback from key informants found that, in all of the workshop sessions, there was a high level of participant engagement and interaction. Additionally, at the conclusion of the study, providers, administrators, and municipal officials all expressed enthusiasm for the training and suggested that the intervention should be included in future facility budgeting activities and scaled throughout the municipality.

At the conclusion of the workshops, representatives from each group came together to generate one unified action plan for the maternity block, including the antenatal, labor, and postnatal wards. This action plan was approved by the hospital management team and integrated into routine facility processes throughout all wards of maternity block. In addition to addressing facility barriers to respectful care, the action plan was designed to empower health care providers and to improve their feelings of self-efficacy and ability to enact change within their workplace. Items in the action plan were contained to activities that staff could conduct on their own, through teamwork and active involvement, without substantial additional resources. One of the primary objectives of the RMC Workshop action plan was to generate conversation about creating a culture of respect at the hospital. Thus the action plan was used as a tool at department meetings, and provided opportunities for staff of all cadres to discuss issues of patient care.

Throughout the implementation period, progress was made on several items from the facility-wide action plan, including:

- A new reporting structure was put in place to speed up the payment of overtime allowances. Before the intervention, overtime payments were often delayed by up to six months; this delay decreased to six weeks by the end of the intervention period.
- Two staff recognition events were held to improve staff motivation. Staff were selected for recognition based on a set list of criteria determined by RMC Workshop participants, including punctuality, number of deliveries conducted, relationships with other staff, team work, and good interpersonal care. Recipients were rewarded with certificates and small gifts, and plans are in place for these events to occur at least annually.
- To improve staff morale, a system was developed to ensure that bread and tea were always available to staff in the break room.
- Issues related to teamwork, communication, provider-patient relationships, and patient rights were discussed weekly at departmental meetings throughout the intervention period.
- Curtains and screens were procured—and existing supplies repaired—to ensure that all beds had a functioning partition to provide privacy.
- To increase the number of staff per shift, nurse shifts were increased from eight to twelve hours. This was initially acceptable to staff, but eventually led to overwork and complaints and the shifts were reversed to eight hours.
- Posters displaying the Universal Rights of Childbearing Women in Kiswahili were displayed in the antenatal and labor wards.
- Staff identified the need to receive feedback from patients more regularly, and a brief and confidential exit survey was designed and piloted in October 2014. The goal of the surveys was to compile feedback quarterly and share with staff during department meetings.
- A brief survey tool was designed to gather staff feedback on supportive supervision, and hospital administrators are continuing to plan methods to improve supportive supervision at the facility.

### Proximal outcomes

#### Patient knowledge

Pre-post tests administered around the Open Birth Day sessions found a notable increase in patient knowledge of many of their rights during labor and delivery (Table 3), including the right to consent (from 30.1 to 57.8 %), the right to be free from physical abuse (from 79.5 to 86.9 %), and the right to privacy (68.2 to 81.9 %). Knowledge of some rights, such as the right to dignified care (from 88.0 to 88.8 %), the right to information (95.1 to 96.7 %), and the right to appropriate and timely care (from 96.4 to 96.2 %), was high at baseline and showed little change. Women’s knowledge of the labor and delivery process also improved through OBD; pre-post tests documented a 13.0 % increase (from 77.8 to 88.0 %) in knowledge of where to check in at the facility when in labor and a 64.7 % increase (from 27.1 to 44.7 %) in knowledge that it is best to move around during labor.

**Table 3:** Changes in patient knowledge (*N* = 362)

Question	Correct Answer	Pre-Test Correct	Post-Test Correct	Percent Change
Any doctor, nurse, or midwife who performs a test on me must ask for my permission first and it is my right to refuse a procedure.	True	30.1	57.8	51.8
It is ok for providers to shout at me, scold me, or say rude things while I am in labor.	False	88.0	88.8	0.9
It is my right to receive care and attention when I need it from a healthcare provider during labor and delivery.	True	96.4	96.2	−0.3
It is acceptable for a health worker to use physical force such as slapping, pinching, or hitting to make me push while in labor.	False	79.5	86.9	9.3
It is my right to privacy, so that my body is not exposed to everyone in the hospital.	True	68.2	81.9	20.1
It is acceptable for a medical staff person to refuse me services, drugs, information, or help based on my religion, age, ethnicity, or wealth.	False	93.4	94.8	1.5
Once a provider says that my baby and I are healthy and ready to be discharged, it is my right to leave the hospital when I want. The hospital cannot make me stay against my will.	True	52.1	52.1	0.0
It is my right to ask for any information about my care and health that I need, including my delivery status, the medication and drugs I am given, and my baby’s health.	True	95.1	96.7	1.7
When I arrive at the hospital, I should check in at __.	Maternity Ward Reception	77.8	88.0	13.0
It is best to sit still and not walk around while I am waiting in the labor ward.	False	27.1	44.7	64.7
A provider will tell me when and how to push with the labor pains (contractions).	True	89.9	93.4	4.0

Provider perspectives of patient preparedness shifted over the course of implementation. During structured provider interviews in the evaluation, 65.5 % of providers said that they could tell whether a woman had attended OBD when she comes to deliver. The most common reasons cited included: the patient knows to bring all emergency supplies she may need (61.1 %), the patient knows where to go for check in and what documents she will need (75.0 %), the patient knows where to wait until she is moved to the next area (69.4 %), and that the woman understands her rights as a patient (86.1 %). Notably, 100 % of providers interviewed during the evaluation who had participated in OBD agreed that the activity prepared women for labor and delivery.

#### Provider knowledge

The intervention package increased aspects of provider knowledge of their code of conduct, ethics, and patient rights. The RMC Workshop pre and post-test data (Table 4) documented a 5.4 % increase in providers who stated that “disrespect and abuse during maternity care is a human rights violation” and a 6.8 % increase in knowledge that disrespect and abuse is a global problem. Further evidence suggests that provider knowledge of informed consent increased, with the RMC Workshop post-tests documenting a 17.3 % reduction in providers who said that it was “safer to withhold information from less educated women.” Two elements of provider knowledge—including the importance of confidentiality and defining communication—decreased slightly from pre- to post-test. Overall, during structured provider interviews during the evaluation, 79.1 % of providers who participated in the RMC Workshop stated that the intervention allowed them to “understand much better” what constitutes patient rights.

**Table 4:** RMC Workshop Pre-Post Tests (*N* = 76)

Question	Pre-Test % Agree	Post-Test % Agree	Percent Change
Provider knowledge			
Disrespect and abuse during maternity care is a human rights violation.	66.7	70.3	5.4
It is safer to withhold information from less educated women who may not understand or become confused and distressed.	53.9	44.6	−17.3
Abusive and disrespectful care occurs in low, medium, and high income countries.	49.3	52.7	6.8
Communication is the ability to build a relationship of trust, understanding, and empathy with the client and to show humanism, sensitivity, and responsiveness.	100.0	95.9	−4.1
Confidentiality is important in family planning and reproductive health care, but not in maternity care.	14.5	17.6	21.5
Provider attitudes			
I have a good understanding of my clients’ backgrounds.	67.1	91.9	36.9
I am able to empathize with my clients.	89.0	98.6	10.8
Clients are always treated respectfully at the Hospital.	48.6	39.7	−18.4
Clients at the Hospital are satisfied with the care they receive.	34.7	18.9	−45.4
I wish to develop stronger relationships with my colleagues at the Hospital.	92.1	100.0	8.6
I believe there is a need for health workers at the Hospital to improve their attitudes towards clients.	100.0	100.0	0.0
As a health worker, I wish to improve the way I treat clients.	98.7	98.6	0.0
Building a strong and cohesive team of health workers is important for delivering high quality maternity care.	98.7	97.3	−1.4
Provider efficacy and empowerment			
As a health worker, it is within my control to provide respectful care to clients.	93.4	93.1	−0.3
If I have a problem at work, I know who I can talk to in order to resolve it.	88.2	91.8	4.1
I have the ability to identify and solve problems in the setting of my work.	80.3	91.7	14.2
I believe my own attitudes can affect the quality of care I provide.	68.4	85.1	24.4
I believe my own attitudes can affect my level of satisfaction with my job.	75.7	83.8	10.7
I believe that change is achievable at the Hospital.	94.5	97.3	2.9
There is nothing I can do to increase my satisfaction with my job.	21.1	10.8	−48.6

#### Patient attitudes and perceptions

Patient attitudes about childbirth at the study facility changed during the intervention period. OBD pre-post tests found that 13.4 % of participants said they felt more comfortable about their upcoming delivery at the study facility after their participation in the program, and the proportion of respondents who said they felt “very comfortable” increased from 67.0 to 73.4 %. Community follow-up interviews four to six weeks after delivery with women who had attended OBD found that 77.8 % of respondents said that participation in OBD made them feel “much more prepared for delivery” while 14.8 % said they felt “somewhat more prepared.” Similarly, 88.9 % of respondents said that the OBD made them feel more comfortable about their delivery at the study facility.

#### Provider attitudes and perceptions

Data from multiple tools indicated that providers’ understanding of their patients’ backgrounds and empathy for their patients increased over time. The RMC Workshop pre-post tests (Table 4) found a 36.9 % increase in providers who said they agreed with the statement “I have a good understanding of my clients’ backgrounds” and a 10.8 % increase in providers who agreed that “I am able to empathize with my clients.” In the evaluation, 75 % of providers who participated in the RMC Workshop “strongly agreed” that the workshop “helped me to improve my interpersonal relationships with clients in the facility.”

Provider attitudes regarding the care provided at the study facility also changed. Results from the RMC Workshop pre-post test indicate that providers’ perceptions of client satisfaction with care at the facility decreased after participation in the workshop (Table 4). Additionally, there was an 8.6 % increase in providers stating that they wished to develop stronger relationships with colleagues and a high percentage (97.3 %) of providers said that building strong teams is important for delivering high quality care. At both pre and post-test, all providers agreed that there is a need to improve attitudes towards clients.

#### Patient-provider communication

During structured provider interviews, 98.2 % said that participation in Open Birth Days improves communication between patients and providers. Additionally, there is evidence that this improved communication continued during labor and delivery as observers noted that providers were more welcoming to women than during baseline and more likely to introduce themselves. Although we did not specifically assess communication between providers and administrators, anecdotal feedback from staff members suggests that the dialogue and communication about respectful maternity care improved. When interviewed during the project evaluation, 78.2 % of providers strongly agreed that their facility has good teamwork between cadres compared with 11 % at baseline.

### Distal outcomes

#### Empowered patients

In addition to being more prepared for delivery and more knowledgeable about their rights, women felt more empowered about their childbirth experience. Nine of the 22 respondents (40.9 %) in the short open-ended interviews with OBD participants remarked that OBD was the first time they had heard anything about their rights during labor and delivery. In particular, two of these respondents noted that they learned for the first time that their consent is required for any procedure and that they can say “no” if/when they do not want or understand a proposed procedure. This increased confidence and knowledge was also manifested in women’s actions. For example, during the baseline assessment, no participant who reported experiencing disrespect and abuse took any actions to rectify or report the situation, while during the evaluation, 10 % of women who attended OBD and reported feeling disrespected during labor and delivery formally filed a complaint.

#### Provider job satisfaction

The RMC Workshop pre-post tests found that providers had increased feelings of efficacy and empowerment (Table 4), including the ability to solve problems at work and a belief that their own attitudes can affect both the quality of care they provide and their job satisfaction. Notably, there was a 48.6 % decrease from pre- to post-test in providers who agreed that there is nothing they can do to improve their job satisfaction.

Structured provider interviews during evaluation showed that 70.8 % of providers who had participated in the RMC Workshop “strongly agreed” that they were better able to communicate with their supervisors about things they would like to change in their role, and 66.7 % said that they were better able to communicate with supervisors about facility-related issues after completing the workshop. Additionally, 65.2 % of providers said that the process of developing and implementing the RMC Workshop action plan changed their perception about how they were able to change facility norms and procedures, and 70.9 % said that the Workshop changed the way they managed job stress.

During structured provider interviews, 41.7 % of providers who participated in the RMC Workshop said that the process of developing and implementing the action plan changed their job satisfaction. As shown in Table 5, many elements of job satisfaction improved over the course of the intervention period. Providers’ described an increased sense of autonomy, improved perceptions of management and supervisors, and improved relationships between staff members.

**Table 5:** Provider job satisfaction (Baseline *N* = 50; Evaluation *N* = 55*)

	Question: "To what extent do you agree that the following are present in your current job?"	Time period	% Strongly agree	% Somewhat agree	% Somewhat disagree	% Strongly disagree
Autonomy	Health professionals control their own practice	Baseline	40.5	40.5	10.8	8.1
		Evaluation	72.7	27.3	0.0	0.0
	There is freedom to make important patient care and work decisions	Baseline	27.8	38.9	30.6	2.8
		Evaluation	72.7	25.5	1.8	0.0
Supervision	A manager who provides supportive supervision and mentorship	Baseline	11.1	52.8	22.2	13.9
		Evaluation	72.7	23.6	0.0	3.6
	A manager who backs up the staff in decision-making and conflict resolution even if the conflict is within cadre, below or with a more qualified member of staff	Baseline	17.1	61.0	12.2	9.8
		Evaluation	74.5	21.8	1.8	1.8
	Adequate clinical supervision	Baseline	28.6	57.1	11.4	2.9
		Evaluation	74.5	18.2	3.6	3.6
	Hospital/clinic managers support and value health workers	Baseline	21.1	42.1	26.3	10.5
		Evaluation	35.2	51.9	3.7	9.3
Teamwork	Doctors, nurses, and other health workers have good working relationships	Baseline	36.8	57.9	5.3	0.0
		Evaluation	67.3	25.5	5.5	1.8
	A lot of teamwork between different cadres of health providers	Baseline	35.1	48.6	13.5	2.7
		Evaluation	78.2	18.2	1.8	1.8

*The number of employed providers at the study facility increased between the baseline assessment and evaluation, accounting for the increased sample size at the time of evaluation

#### Improved patient-provider interactions

The package of interventions also had an effect on the quality of patient-provider interactions. Women’s responses during short open-ended interviews after OBD sessions indicate that participants were able to view and interact with providers “as people” (verbatim term used by women), likely as a result of having a venue to engage with providers in a more informal manner with more opportunities for dialogue than the formal antenatal care visit. A few participants interviewed commented that they liked the way that nurses provided education and spoke to them during these sessions, with one woman saying the most important thing she learned at OBD sessions was about “the good collaboration of the nurses” with patients.

Additionally, providers indicated that they felt an increased responsibility after the interventions to provide high quality, respectful care to their patients. All providers (*n* = 25) who were interviewed at evaluation and had participated in the RMC Workshop reported that the Workshop changed the way they thought about and interacted with their patients. When asked to elaborate, provider responses included:

- “It [RMC Workshop] has helped, it reminded us to provide the services they need.”
- “I have managed to keep myself good/updated because I must give mother her rights, I might give birth at the hospital too so the rights should be adhered to.”
- “How to talk to patients with love together with listening to the client.”
- “Because I know clients’ rights and she is aware of her rights, too, there are significant changes.”
- “Using good language brings peace and joy to the patients.”

#### Patient satisfaction and perceptions of quality

**Fig. 2:**
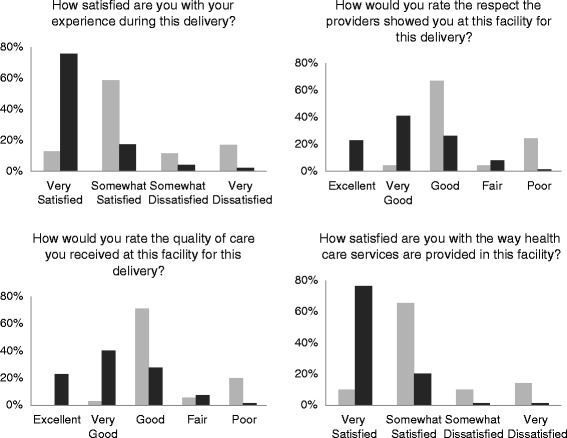
Patient perceptions of satisfaction with delivery and health care services, quality of care, and provider respectfulness. *Light gray bars* indicate baseline community follow-up responses (*N* = 70) and *dark gray* indicate evaluation community follow-up responses (*N* = 149)

Patient satisfaction with services received and their perceptions of quality improved substantially from baseline. During community follow-up interviews, 75.8 % of women reported being very satisfied with their delivery experience compared to only 12.9 % at baseline (Fig. 2). Similarly, at the time of evaluation, 22.8 % of women rated the respect shown to them by providers as “excellent” compared to none at baseline. At baseline, no women rated the quality of care they received as “excellent” and only 2.9 % gave a “very good” rating; at the time of evaluation, these frequencies increased to 22.8 and 40.3 %, respectively. Finally, patient satisfaction with the way that health care services were provided at the study facility also improved, with 76.5 % of women reporting “very satisfied” at the evaluation compared to 10.0 % at baseline.

## Discussion

This paper is one of the first to describe in detail interventions designed to mitigate disrespect and abuse, including a description of the theory of change, reporting of the implementation process, and the provision of detailed and relevant process data that can be used to guide replication and scale-up efforts [19, 26]. While we were not able to rigorously evaluate the impact of the interventions, we were able to carefully monitor the process and explore its effects on proximal and distal outcomes (Fig. 1).

Overall, the results of this study indicate substantial positive changes in providers and patients, which we consider promising. Women who participated in Open Birth Day increased their knowledge of their own rights and felt empowered during labor and delivery. Women’s increased reporting of D&A to facility officials particularly highlights this effect. Although only 10 % of women who experienced D&A lodged a formal complaint, this is an important shift from baseline when no women who reported experiencing D&A took any follow-up actions. This behavior is also indicative of a change in facility norms towards increased accountability.

After the interventions, providers had increased knowledge of patient rights, a greater capacity to empathize with the women they serve, and improved job satisfaction. However, not all elements improved to the same degree. For example, slight decreases in the percent of providers who stated that confidentiality is important for maternity care and in those who identified critical interpersonal aspects of communication may indicate that not all topics were covered successfully by the RMC Workshop, or that some aspects of provider knowledge would require a different intervention to improve. Notably, however, the RMC Workshop pre-post tests demonstrated that providers were able to more realistically and honestly evaluate the care provided at the study facility and the level of patient satisfaction. Additionally, providers demonstrated a marked desire to improve the care they and the facility provide, as well as an increased belief that changes within the facility are possible.

A recent study in Kenya employed, among other interventions, activities similar to Open Birth Days and the RMC Workshop and achieved a 7 % reduction in reports of disrespect and abuse [19]. Although an impact evaluation was beyond the scope of our project, we can hypothesize that our interventions also had a positive impact on mitigating disrespect and abuse. Unfortunately, our evaluation design and sample size did not allow us to demonstrate this potential effect (see below).

The theory of change employed by this study targeted three proximal outcomes and four distal outcomes as proxies or intermediate steps towards reducing disrespect and abuse. These outcomes were selected based on extensive literature reviews [11]; discussions with experts in the respectful maternity care field; interviews with key stakeholders in Tanzania; and the feedback received from patients, providers, administrators, and policy-makers throughout the baseline dissemination process. Our comprehensive process allowed us to identify and measure outcomes that were relevant for this setting, but we recognize that additional or alternative outcomes may be pertinent in other contexts, depending on the local contributory factors to disrespectful and abusive behaviors. Additionally, the interventions were selected to be effective in our particular study facility. The interventions, in their current form, may be more or less applicable to different contexts. In order to advance the body of knowledge around effective and efficient means of promoting respectful maternity care, we strongly recommend that other studies conducted in this field make explicit their theories of change and monitoring processes to facilitate interpretation of findings, and replication and scale-up of effective interventions.

This study had several limitations. The pre and post-test instruments used in both OBD and the RMC workshop were not validated, while the observation, provider interview, and patient interview instruments were adapted from tools that were validated in Kenya but not in Tanzania [26]. A Hawthorne effect is possible, as the implementation and evaluation phases of the study overlapped; therefore, provider and facility-level behaviors may have been impacted by the knowledge that an assessment was ongoing. The study team was an active partner in the identification and implementation of the interventions described here; this was a critical enabler of the successes demonstrated but has implications for the repeatability and generalizability of these interventions in the future or beyond the study setting. Based on our experience, we strongly recommend using a participatory process to identify acceptable, sustainable and appropriate interventions for addressing disrespect and abuse.

Finally, a critical limitation of this exploratory study is the lack of a rigorous evaluation. This study sought to and was successful in exploring the prevalence and drivers of disrespect and abuse in a large public hospital in Tanzania, engaging with partners to identify feasible and acceptable interventions, and exploring the effects of these interventions on patients and providers. However, in order to reach firm conclusions about the impact of these interventions, a study with a more rigorous design and enough power to establish the statistical significance of the changes needs to be undertaken.

## Conclusions

The results presented here indicate that health facility staff, authorities and administrators can be successfully engaged in the identification, development and implementation of acceptable, feasible and low-cost interventions that have an effect on upstream predictors of disrespect and abuse. The interventions described here ranged from small structural changes, such as the addition of privacy curtains to delivery beds, to more complex behavior change and values clarification activities like the RMC Workshop. From our perspective, the complex network of factors that contribute to disrespect and abuse during labor and delivery require a multipronged approach, like the one used in this study. The sustained presence of project staff in the facility, working in close collaboration with facility leaders, allowed for the coordinated delivery of these multifaceted efforts. The data presented here indicate that significant progress was made towards changing the context in which care is delivered at the study facility, although some infrastructure challenges and shortages, such as bed space, persisted and will require increased and sustained investment and commitment to address. Further evaluation efforts are needed to assess the impact of the interventions on disrespect and abuse, and monitor the sustainability of these changes over time.
